# A Missense Mutation in LMX1A in a Patient With Moebius Syndrome: A Case Report

**DOI:** 10.7759/cureus.30127

**Published:** 2022-10-10

**Authors:** Ghaliah O Alnefaie

**Affiliations:** 1 Department of Pathology, College of Medicine, Taif University, Taif, SAU

**Keywords:** case report, missense mutation, p.gln61arg, lmx1a, moebius syndrome

## Abstract

Moebius syndrome is characterized by congenital complete or partial paralysis of the facial nerve and is often associated with orofacial and limb malformations. It is a rare syndrome that affects the sixth and seventh cranial nerves. Facial paralysis results in abnormal abduction of one or both eyes and facial paralysis or weakness. Moebius syndrome is an uncommon condition and only a few hundred cases have been reported in the literature. A seven-year-old girl with Moebius syndrome is featured in this report. She had asymmetrical facial expressions, ocular abduction anomalies, and swallowing difficulties. She also had mild low-set ears, hypertelorism, a short nose, and restricted jaw movements. Array-comparative genomic hybridization analysis of exosome sequencing showed a mutation p.Gln61Arg in exon 3 of *LMX1A*.

## Introduction

Moebius syndrome (MBS), a rare congenital disease, was first described by Von Graefe in 1880 and then named after Moebius in 1888 [1]. It is characterized by unilateral or bilateral facial and abducens palsy, which leads to a loss of facial expressions, and an inability to smile or swallow [1]. Moreover, patients with MBS also suffer from orofacial and limb defects. The estimated prevalence is 1/250,000, with an equal incidence in both genders [2]. The condition affects one in 50,000 to one in 500,000 newborns [3]. The unclear diagnostic criteria have led to difficulty in the clinical assessment, prognosis, and genetic analysis of patients with MBS. Since 1888, the pathogenesis of the disease has been unclear; till 2007 when MBS was defined as “congenital unilateral or bilateral, non-progressive facial weakness and limited abduction of the eye” [2].

Furthermore, *de novo* mutations in *the PLXND1* and *REV3L* have been recorded in many MBS cases. *PLXND1* plays a role in regulating the migration of a wide spectrum of cell types in the striatum and is selectively expressed in direct-pathway medium spiny neurons [4]. Although *REV3L* functions in the translation of DNA synthesis, it can also protect DNA from damage [5].

## Case presentation

**Figure 1:**
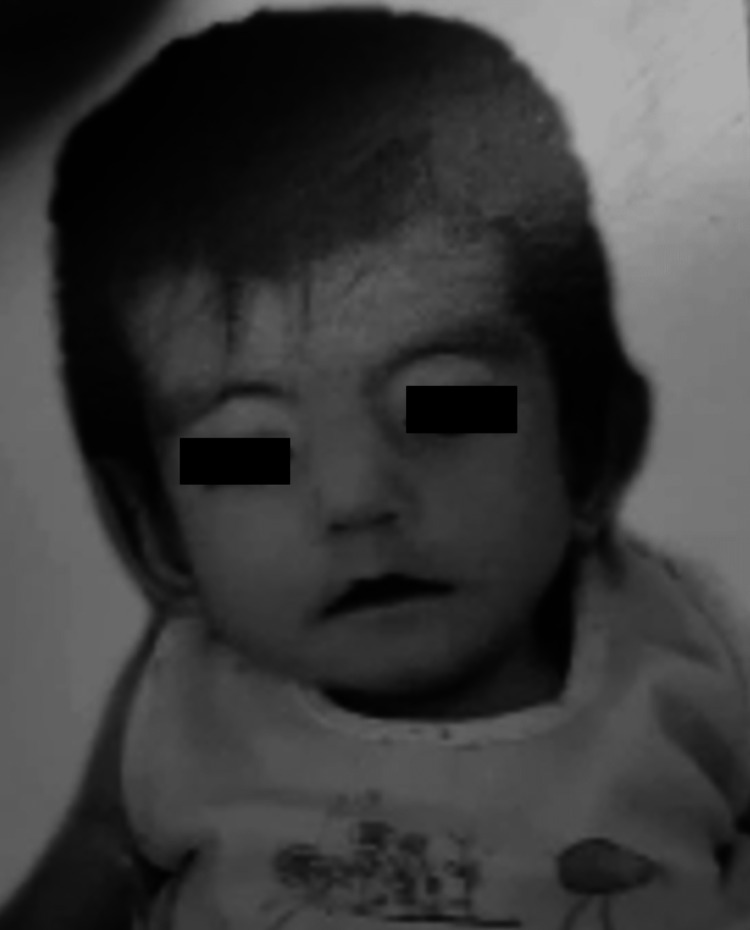
Photo of the patient taken at age of 15 months showing dysmorphic feature hypotonia, bilateral epicanthic, and a congenital facial weakness

A seven-year-old female experienced global developmental delay and weakness of the facial muscles. On examination: the patient had dysmorphic features such as hypotonia and bilateral epicanthal fold and was born with a small chin (micrognathia), a broad nasal bridge, and a small mouth (microstomia) with an unusually shaped tongue. She had difficulty making eye contact, her eyes were misaligned (strabismus), and she exhibited a lack of facial expression, including smiling, frowning, raising eyebrows, puckering lips, and closing her eyes (Figure 1).

She also exhibited a weak muscle tone (hypotonia). In general, the patient experienced delayed development of motor skills (such as crawling and walking), difficulties with speech, frequent drooling, and difficulty swallowing or sucking. She also had a low-set ear, hypertelorism, a short nose, a short philtrum, a carpal moth, and restricted jaw movement (Table 1). Mild puffiness of the cheeks and mild asymmetry was observed during unsupported walking. Dental abnormalities included missing and misaligned teeth which contributed to speech difficulties. All clinical features mentioned earlier indicated MBS.

**Table 1:** Features of Moebius syndrome and their presence in our patient

Moebius syndrome features present in our patient's clinical appearance					
Dysarthria	Yes	Ears dysplastic or low set	Yes	Upper limb anomaly	Yes
Abnormal motor coordination	Yes	Hypoplastic tongue	Yes	Talipes	Yes
Failure to thrive	Yes	High palatal arch	Yes	Brachydactyly	No
Micrognathia	Yes	Blepharoptosis	Yes	Poland anomaly	No
Hypotonia	Yes	Cleft palate	No	Syndactyly	No
Epicanthic folds	Yes	Cardiac anomalies	No	Scoliosis	No
Developmental delay	Yes	Conductive deafness	No	Symbrachydactyly	No
Swallowing difficulties older	Yes	Limb and skeletal anomalies	Yes	Camptodactyly	No
Brain imaging abnormality	No	Lower limb anomaly	Yes		

Diagnostic assessment

Very long-chain fatty acids (VLCFA) testing was performed due to the matching of many of the clinical features of the case above with the known features of Zellweger syndrome including dysmorphic facies, hearing loss, and hypotonia. The test result came back normal, so Zellweger syndrome was ruled out. Her metabolic screening and toxoplasmosis, rubella cytomegalovirus, herpes simplex, and HIV (TORCH) titers were normal, with no cherry-red spot-on ophthalmology, which may be present in many lysosomal storage diseases. Electroencephalography (EEG) showed no abnormal electric activity that may point out possible seizures.

Brain and abdominal ultrasound findings were normal. Moreover, magnetic resonance imaging (MRI) of the brain showed increased intensity at the lentiform nucleus, mainly at globus pallidus initially; however, on follow-up, MRI revealed a significant reduction of the signals observed earlier in the basal ganglia. Hydrocephalus was observed. Her results for tandem mass spectrometry and lateral flow test for blood gases were all within normal levels. The rest of the blood tests were normal including haematology and chemistry. (Table 2).

**Table 2:** Timeline summarising the multiple hospitalisations and clinical referrals of the patient SVD: spontaneous vaginal delivery; CBC: complete blood count; TORCH: toxoplasmosis, rubella cytomegalovirus, herpes simplex, and HIV; DDH: developmental dysplasia of the hip; GVS: galvanic vestibular stimulation; BCG: bacille Calmette-Guerin; PICU: pediatric intensive care unit; FISH: fluorescence in situ hybridization; VLCFA: very long-chain fatty acids; TMS: transcranial magnetic stimulation; CGH: comparative genomic hybridization; CARS: childhood autism rating scale

Dates	Relevant Past Medical History and Interventions		
	Preterm 35 weeks, respiratory distress syndrome, hypotonic, congested face to rule out sepsis, dysmorphic; Maternal history: G 10 P 5+4, un-booked, mitral valve prolapse on propranolol, no consanguinity, Birth history: spontaneous vaginal delivery SVD, routine resuscitation, Apgar score 8 and 9 at one and five minutes, congested face and hypotonic. Birth weight: 2.625 kg head circumference: 33 cm, length:49 cm.		
Dates	Clinical history and presenting complaint of each visit	Diagnostic Testing	Interventions
Date of admission 5.4.2015, two weeks PICU; five months hospitalised; date of discharge: 16.8.2015	Physical examination: hypnotic, congested face, recessed mandible apparently slants, broad nasal bridge and short philtrum partial ptosis Gastrostomy tube passed on 26.7.2015	CBC: normal; Chemistry: normal; Metabolic screening: normal EEG: normal, no seizures activity MRI: increased intensity at lentiform nucleus mainly at globus pallidus initially, but on follow-up MRI revealed a signal effect on basal ganglia is reduced significantly; Cranial ultrasound: normal; TORCH titer: normal; Hips: no evidence; DDH Abdominal: soft and lax, no organomegaly; GVS: S1+ S2+0; Respiratory: Bilateral grossly clear chest with equal air entry; Eye: no cheesy red spot, partial ptosis periodic upward rolling of eyeballs; CNS: central hypotonic. Baby is floppy: with head lag, tone and reflexes were normal but the baby is floppy (floppy strong)	Medication: tonics; vaccination: BCG and hepatitis B vaccine 1st dose given Feeding: Gastrostomy tube feeding (122 cal/kg/daily); Family education: provided
Paediatric department 19.10.2016	Two-year-old Saudi female presented to the clinic for first time with central hypotonia, dysmorphic feature, poor sucking, developmental delay, and no seizure.	Karyotype study: normal; FISH study for Prader-Willi Syndrome: negative; VLCFA: normal TMS, an ammonia levels test NH3, Liver function tests LFT, blood gases: all normal level	Physiotherapy has started CGH/ exome sequencing has been required
Paediatric department 4.7.2018	3-year-old girl who is followed in the genetic clinic for global development delay, and facial dysmorphic. Facial dysmorphism with Moebius sequence ptosis hypertelorism short nose short philtrum carpal mouth low set ears. She can stand and walk with support. Delayed physical development, sitting at two years, walking at 3½ years, and sphincter control not until 2½ years. Gastrostomy tube removed	Global development delay, facial dysmorphic and Moebius sequence caused by *LMX1A* mutation Karyotype: 46, XX, normal. Whole exome sequencing: the presence of heterogeneous mutation P.Q61R at exon 3 of *LMX1A* gene. Brain MRI: normal. Abdomen ultrasound normal.	Referring to the audiology clinic
Audiology clinic 15.11.2018	Delayed language development and inconsistent responses to sounds. Negative sanguinity. Negative family history of hearing loss and delayed language development. Right, type (C) left type (B) tympanogram. Acoustic reflexes, bilateral lost	Audiometry showed: The hearing threshold level is about 40dBnHL. left severe mixed hearing loss. The hearing threshold level is about 70dBnHL.	Treatment of right Eustachian tube dysfunction and left middle ear effusion. Hearing aids, psychometric evaluation, the CARS test, speech training, instruction list, follow-up, and retest after treatment
At six years	Since birth had problems with swallowing unable to speak. There was motor retardation with improvement in the last four months. Bilateral ptosis, strait gaze, not able to move eyes, no facial expression. Was able to swallow but slowly. Low-set ears. Jaw movement is restricted. There is also mild underdevelopment of cheeks and mild asymmetry there is obvious palsy of VI and VII cranial bilateral, but glossopharyngeal nerve palsy might be also present considering swallow problems.		Might be part of Moebius syndrome Considering this possibility, child be referred to audiology for VII cranial palsy.

**Figure 2:**
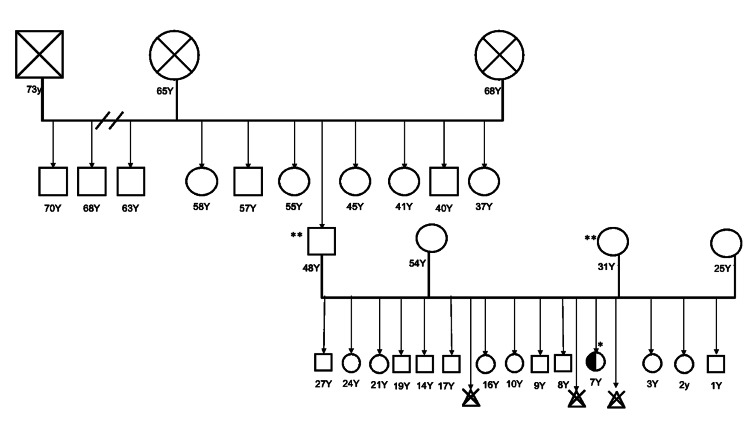
Three-generation pedigree showing that the P.Q61R mutation in exon 3 of LMX1A presented only in the affected girl; it is a de novo mutation. Whole exome sequencing *Showing a heterogeneous p.Gln61Arg mutation in exon 3 of *LMX1A* **Not showing p.Gln61Arg mutation in exon 3 of *LMX1A*

She had non-consanguineous parents who had three abortions (Figure 2), with no deaths or similar conditions. The normal female karyotype 46 XX and Prader-Willi/Angelman (15q11-13) duplication in *SNRPN* were negative.

Whole exome sequencing showed a heterogeneous p.Q61R mutation in exon 3 of *LMX1A*. A de novo heterozygous missense variant c.182A>G (p.Gln61Arg) in the *LMX1A* gene is not reported by GnomAD. In addition, a whole exome analysis was also conducted for parents in order to determine whether the variation in *LMX1A* was inherited from the parents or whether it was a de novo event. Due to this, neither parent has c.182A>G, suggesting that it is de novo. In addition, the *PLXND1* and *REV3L* genes, which are related to Moebius syndrome, were investigated to determine whether there is any gene defect that contributes to patient characteristics. However, no mutations were identified.

Therapeutic intervention

The patient’s hearing dysfunction and left middle ear effusion were managed using a eustachian tube. Her vision was treated with supporting glasses, and she underwent speech therapy with a specialist. There is no specific treatment course for Moebius syndrome. The treatment, in this case, was supportive and in accordance with the symptoms that were presented. Infants may require feeding tubes or special bottles to provide sufficient nutrition. In this case, the patient had a gastrostomy tube placed at birth, which was removed later at age of three years. As a part of surgical treatment, tarsorrhaphy and smile surgery can be performed later in life. Physical and speech therapy often improves motor skills and coordination and leads to better control of speaking and eating abilities. Plastic reconstruction surgery may be beneficial, and nerve and muscle transfers to the corners of the mouth can be performed to provide a limited ability to smile.

Follow-up and outcomes/patient perspectives

The patient was recommended to attend the follow-up to make a general check-up to assess the physical, vision and hearing progress and to visit a dentist clinic. The physical progress has been appreciated by the patient and her parents. That progress comes from parent support by keeping their faith and constantly attending the follow-up session.

## Discussion

Genetic abnormalities in most MBS cases are unclear, as they generally show sporadic mutation [6]. However, a familiar trend has been indicated in patterns of both autosomal dominant and recessive inheritance [7]. Moreover, the inherent genetic risk ranges between 2% and 30% according to clinical features [4]. The risk is reduced to 2% if MBS affects only the musculoskeletal system [5]. However, if MBS presents facial palsy, deafness, and abduction limitation of the eye, the risk increases to 25-30% [5]. Studying the genetics of MBS cases is difficult because they are heterogeneous. Many genetic studies have been conducted to investigate the specific locus in selected genes (1p22, 3q21-22, 10q21.3-q22.1, and 13q12.2-q). The selected loci indicated MBS1 at 13q12.2-q1311 [8]. The loci selected for investigation are also linked to homeobox genes (*HOXA1* and *HOXB1*) and SRY-box transcription factor 14 (*SOX14*), which are critical for brain development. Verzijl et al. defined MBS3 as 10q21.3-3q22.1, in a Dutch family [9]. Some of the investigated genes are important in neural development (*PLEX1N-A1, GATA2, EGR2, BASP1,* and *TUBB3*) [10] and remain linked to growth; for example, *FLT1/VEGFR1* [11]. The most noticeable sporadic mutations reported in six MBS cases were found in *PLXND1* and *REV3L*, which are responsible for facial branchiomotor neuron migration and craniofacial bone abnormality [12].

In this case report, a mutation in *LMX1A* was identified in a patient with MBS. Encoding a protein, *LMX1A* is a transcription factor that positively regulates insulin gene transcription. It also plays a role in the development of dopamine-producing neurons during embryogenesis. However, a mutation in *LMX1A* is associated with an increased risk of developing Parkinson’s disease [13]. The point mutation (c) that occurred in the patient’s chromosome is heterozygous missense variant c.182A>G (p.Gln61Arg).

One study identified that the heterozygous missense variant c.182A>G (p.Gln61Arg) leads to non-syndromic hearing impairment and vestibular dysfunction [14], which points to one of the features of MBS. Furthermore, *LMX1A* has been reported to play a role in the development and specification of dorsal cell fates in the central nervous system and in developing vertebrae [15] which may be linked to weakness of palsy of multiple cranial nerves in MBS, which most patients with MBS experience, including the patient in this case. Several SNPs of the *LMX1A* gene have been found to be associated with congenital scoliosis and other forms of congenital malformations phenotypes of congenital scoliosis [16].

At present, only deafness, autosomal dominant 7 (OMIM#601412), is associated on the Online Mendelian Inheritance in Man (OMIM™) website. Though this variant is absent in the Genome Aggregation Database (gnomAD), it has been predicted to be benign by various in silico tools. More importantly, variants in the *LMX1A* gene were reported to be related to autosomal dominant deafness. The relationship between the *LMX1A* gene and Moebius syndrome has not been well established.

## Conclusions

Although MBS has been reported in several studies, the genetic etiology remains unclear. Efforts are ongoing to recognize the genetic mutations in all MBS cases to characterize a clear guide for the mechanism and causes of Moebius syndrome. Therefore, this case report helps enrich this field with a new aspect.

## References

[1] Von Graefe A, Saemisch T (1877). Manual of Total Ophthalmology (Book in German). Saemisch. Leipzig: W. Englemann. 1880.

[2] Miller G (2007). Neurological disorders. The mystery of the missing smile. Science.

[3] Carta A, Favilla S, Calzetti G (2021). The epidemiology of Moebius syndrome in Italy. Orphanet J. Rare Dis.

[4] Ding JB, Oh WJ, Sabatini BL, Gu C (2011). Semaphorin 3E-Plexin-D1 signaling controls pathway-specific synapse formation in the striatum. Nat Neurosci.

[5] MacDermot KD, Winter RM, Taylor D, Baraitser M (1991). Oculofacialbulbar palsy in mother and son: review of 26 reports of familial transmission within the 'Möbius spectrum of defects'. J Med Genet.

[6] Yaqoob A, Dar W, Raina A (2021). Moebius syndrome. Ann Indian Acad Neurol.

[7] Lehky T, Joseph R, Toro C (2021). Differentiating Moebius syndrome and other congenital facial weakness disorders with electrodiagnostic studies. Muscle Nerve.

[8] Slee JJ, Smart RD, Viljoen DL (1991). Deletion of chromosome 13 in Moebius syndrome. J Med Genet.

[9] Verzijl HT, van den Helm B, Veldman B, Hamel BC, Kuyt LP, Padberg GW, Kremer H (1999). A second gene for autosomal dominant Möbius syndrome is localized to chromosome 10q, in a Dutch family. Am J Hum Genet.

[10] van der Zwaag B, Hellemons AJ, Leenders WP, Burbach JP, Brunner HG, Padberg GW, Van Bokhoven H (2002). PLEXIN-D1, a novel plexin family member, is expressed in vascular endothelium and the central nervous system during mouse embryogenesis. Dev Dyn.

[11] Kadakia S, Helman SN, Schwedhelm T, Saman M, Azizzadeh B (2015). Examining the genetics of congenital facial paralysis--a closer look at Moebius syndrome. Oral Maxillofac Surg.

[12] Tomas-Roca L, Tsaalbi-Shtylik A, Jansen JG (2015). De novo mutations in PLXND1 and REV3L cause Möbius syndrome. Nat Commun.

[13] Bergman O, Håkansson A, Westberg L (2009). Do polymorphisms in transcription factors LMX1A and LMX1B influence the risk for Parkinson's disease?. J Neural Transm (Vienna).

[14] Wesdorp M, de Koning Gans PA, Schraders M (2018). Heterozygous missense variants of LMX1A lead to nonsyndromic hearing impairment and vestibular dysfunction. Hum Genet.

[15] Millonig JH, Millen KJ, Hatten ME (2000). The mouse Dreher gene Lmx1a controls formation of the roof plate in the vertebrate CNS. Nature.

[16] Wu N, Yuan S, Liu J (2014). Association of LMX1A genetic polymorphisms with susceptibility to congenital scoliosis in Chinese Han population. Spine (Phila Pa 1976).

